# A method for labelling lesions for machine learning and some new observations on osteochondrosis in computed tomographic scans of four pig joints

**DOI:** 10.1186/s12917-022-03426-x

**Published:** 2022-08-31

**Authors:** Kristin Olstad, Lars Erik Gangsei, Jørgen Kongsro

**Affiliations:** 1 Faculty of Veterinary Medicine, Department of Companion Animal Clinical Sciences, Equine Section, Norwegian University of Life Sciences, P.O. Box 5003, 1432 Ås, Norway; 2 Animalia AS, Lørenveien 38, 0585 Oslo, Norway; 3 Faculty of Chemistry, Biotechnology and Food Science, Bioinformatics, Norwegian University of Life Sciences, P.O. Box 5003, 1432 Ås, Norway; 4 Norsvin SA, Storhamargata 44, 2317 Hamar, Norway

**Keywords:** Articular osteochondrosis, Breeding selection, Helical computed tomography, Lesion number, Lesion volume, Machine learning, Swine

## Abstract

**Background:**

Osteochondrosis is a major cause of leg weakness in pigs. Selection against osteochondrosis is currently based on manual scoring of computed tomographic (CT) scans for the presence of osteochondrosis manifesta lesions. It would be advantageous if osteochondrosis could be diagnosed automatically, through artificial intelligence methods using machine learning. The aim of this study was to describe a method for labelling articular osteochondrosis lesions in CT scans of four pig joints to guide development of future machine learning algorithms, and to report new observations made during the labelling process. The shoulder, elbow, stifle and hock joints were evaluated in CT scans of 201 pigs.

**Results:**

Six thousand two hundred fifty osteochondrosis manifesta and cyst-like lesions were labelled in 201 pigs representing a total volume of 211,721.83 mm^3^. The per-joint prevalence of osteochondrosis ranged from 64.7% in the hock to 100% in the stifle joint. The lowest number of lesions was found in the hock joint at 208 lesions, and the highest number of lesions was found in the stifle joint at 4306 lesions. The mean volume per lesion ranged from 26.21 mm^3^ in the shoulder to 42.06 mm^3^ in the elbow joint. Pigs with the highest number of lesions had small lesions, whereas pigs with few lesions frequently had large lesions, that have the potential to become clinically significant. In the stifle joint, lesion number had a moderate negative correlation with mean lesion volume at *r* = − 0.54, *p* < 0.001.

**Conclusions:**

The described labelling method is an important step towards developing a machine learning algorithm that will enable automated diagnosis of osteochondrosis manifesta and cyst-like lesions. Both lesion number and volume should be considered during breeding selection. The apparent inverse relationship between lesion number and volume warrants further investigation.

**Supplementary Information:**

The online version contains supplementary material available at 10.1186/s12917-022-03426-x.

## Background

Osteochondrosis is defined as a focal disturbance in endochondral ossification that is due to a failure of the blood supply to growth cartilage [1, 2]. Growth cartilage is typically found in two locations at the ends of long bones: in the metaphyseal growth plate or physis located between the primary, diaphyseal and the secondary, epiphyseal centres of ossification, and in the epiphyseal growth cartilage located between the secondary ossification centre and the articular cartilage [2]. Epiphyseal growth cartilage has a temporary blood supply that tends to be organised as anatomical end arteries, coursing in and out of the cartilage via the same, blind-ending canal [3, 4]. Failure of the blood supply leads to ischaemic necrosis of chondrocytes in the growth cartilage, resulting in a lesion known as osteochondrosis latens [3, 4]. With time, the ossification front advances to surround the area of ischaemic chondronecrosis, which becomes apparent as a focal delay of ossification, creating a lesion known as an osteochondrosis manifesta [4–6]. From this stage, when osteochondrosis lesions become outlined by bone, they are detectable in computed tomographic (CT) scans [7]. Osteochondrosis lesions can resolve spontaneously, or they can progress to osteochondrosis dissecans (OCD), characterised by the presence of fragments in joints, or to subchondral bone cysts [6–8]. Osteochondrosis lesions that persist can also result in development of irreversible, debilitating osteoarthritis [1, 9].

Osteochondrosis and osteoarthritis are the two main causes of lameness or “leg weakness” in pigs [10], and locomotor problems are one of the most common reasons for premature culling of breeding sows [11]. Osteochondrosis is moderately heritable [12], and it is therefore necessary to score and select against osteochondrosis in commercial pig production. Historically, a breeding value was estimated for prospective breeding boars based on pedigree and macroscopic scoring of osteochondrosis in half-carcasses of related pigs in the slaughterhouse. In 2008, a breeding company started using whole-body CT of sedated boars for routine quantification of lean meat and fat percentage for selection [13], and with that, it became possible to score osteochondrosis in CT images of joints in the prospective breeding boar itself, as well as in its relatives [12]. At present, osteochondrosis is scored manually on a scale from 0 to 5 in eight regions: medially and laterally in the left and right elbow and stifle joints [12]. Manual scoring is effective, but time-consuming and it would therefore be better if osteochondrosis could be diagnosed automatically, through artificial intelligence methods using machine learning [14]. In the context of osteochondrosis, machine learning requires a set of CT scans that have been labelled (synonyms: tagged, annotated) by a radiologist who recognises the difference between normal features and lesions. The current study was motivated by the need to produce such a set of labelled CT scans. To match the currently used manual scoring, automated diagnosis must work in the elbow and stifle joints [12]. The hock joint was also included in the current study, because it is known to have a high incidence [8] and prevalence of osteochondrosis [15], thus automated diagnosis could become relevant in this joint in future. The shoulder joint was included mainly because it represents a joint within the forelimb that has a longer growth period than the elbow [16]. Multiple breeds were included to ensure that future automated diagnosis would work in more than one breed of pig.

In summary, our study aims to describe a method for labelling articular osteochondrosis lesions in CT scans of four pig joints for future machine learning, and report any new observations made during the labelling process.

## Results

### Pigs and joints

Data for 201 pigs were extracted, comprising 52 Landrace boars, 49 Duroc boars, 50 Synthetic A and 50 Synthetic B boars. The pigs were numbered 1–201 in random order. At the time of CT-scanning, the pigs had a mean age of 161.5 days (SD 10.3) and a mean weight of 121.3 kg (SD 12.5). All shoulder, elbow, stifle and hock joints were fully visualised except that only the proximal half of the talus was evaluated in 8/402 hocks (2%) because the distal half was outside the collimated field of view.

### Qualitative description of the labelling process – stifle joint

**Fig. 1:**
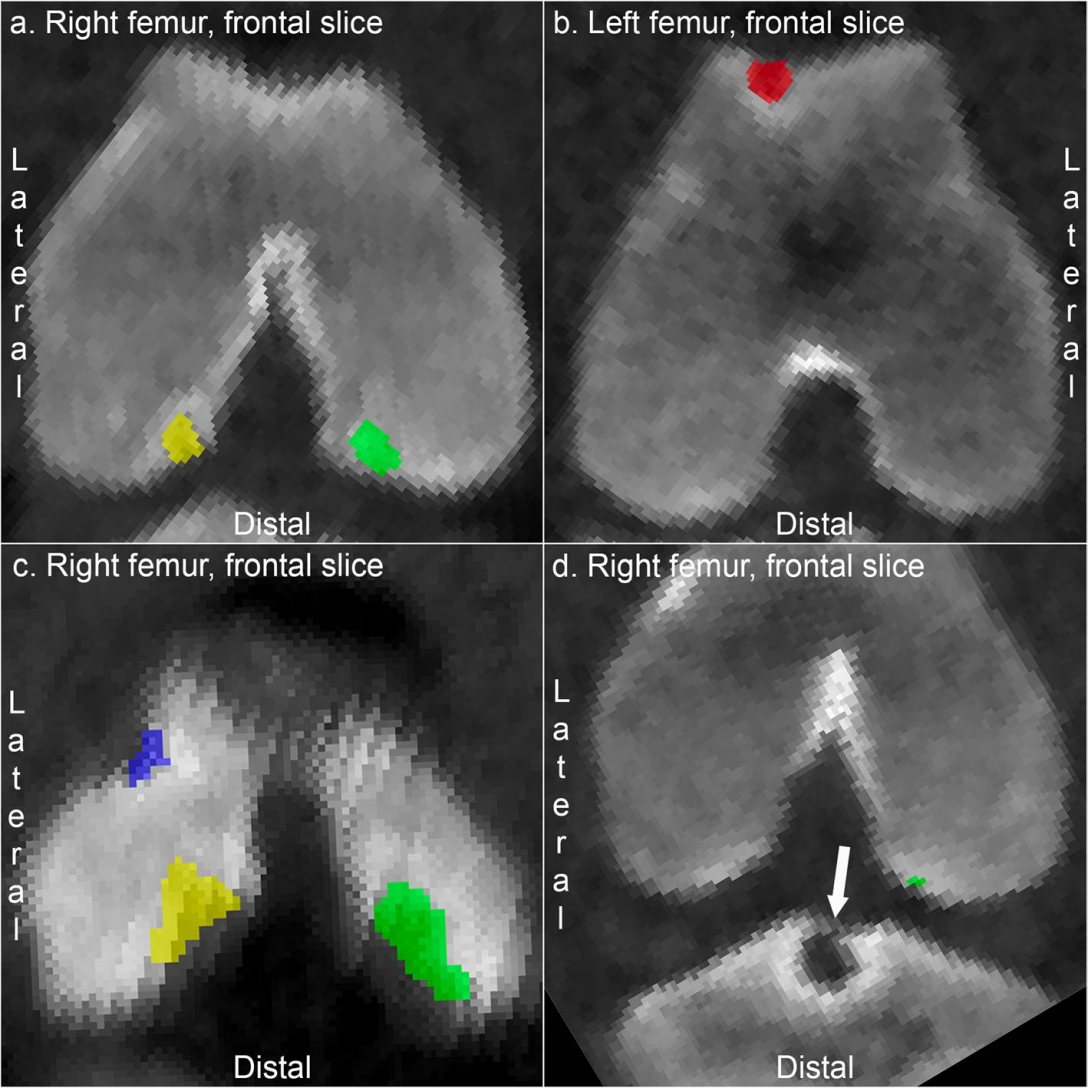
Labelling of lesions in the stifle joint. **a** Pig 3. An osteochondrosis lesion is labelled yellow in the lateral femoral condyle, and a cyst-like osteochondrosis lesion is labelled green in the medial femoral condyle. **b** Pig 4. A cyst-like lesion is labelled red in the medial trochlear ridge. **c** Pig 26. There is an osteochondrosis lesion at the extensor fossa that was labelled blue and grouped with lateral trochlear lesions, and osteochondrosis lesions labelled in yellow in the lateral and green in the medial condyle, respectively. **d** Pig 21. The arrow points to a large, cyst-like lesion immediately deep to the intercondylar eminences of the tibia that is not labelled because the labelling application did not include any tibial regions. There is also a small osteochondrosis lesion labelled green in the medial femoral condyle

The majority of changes observed in the stifle joint were restricted to the four anatomical regions included in the labelling application (see Methods) and could be labelled as lesions (Figs. 1a-c). Trochlear lesions (Fig. 1b) were sometimes located in the trochlear groove, in which case they were labelled as medial or lateral according to where the bulk of the lesion was centred. Lateral lesions included lesions at the trochlear ridge, condyle and extensor fossa predilection sites (Fig. 1c). The latter tended to be located towards the trochlear side of the fossa and were therefore grouped with trochlear lesions. Unforeseen changes were detected in a low number of stifles that could not be labelled as lesions because they were in the proximal tibia (Fig. 1d), and the labelling application did not include any tibial regions. The changes consisted of large, cyst-like lesions immediately deep to the intercondylar eminences of the tibia (Fig. 1d), near where cruciate and meniscal ligaments attach.

Movies of two- and three-dimensional illustrations of labelling of osteochondrosis lesions in the distal femur are available in Supplemental Movies 1 and 2.

### Shoulder joint

**Fig. 2:**
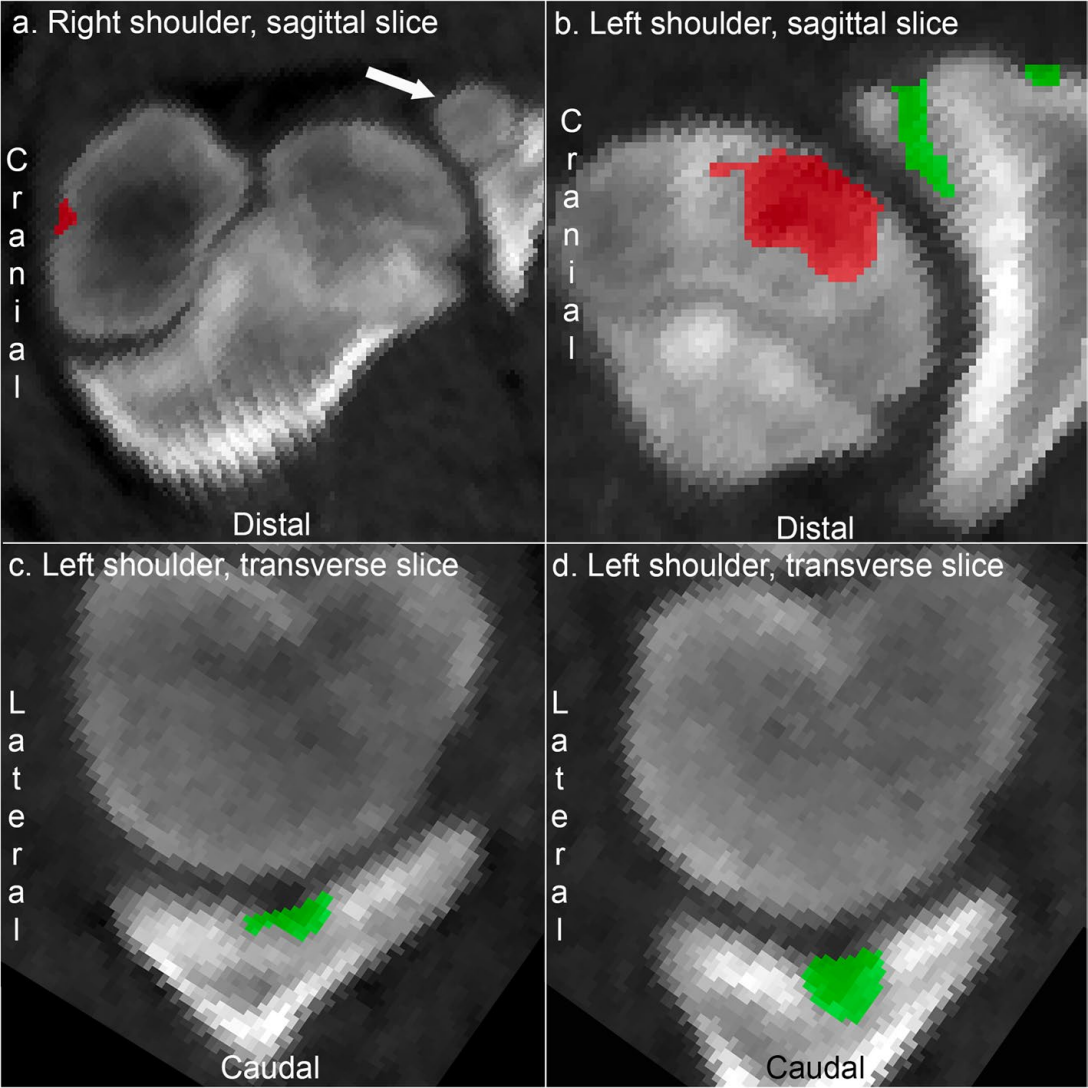
Labelling of lesions in the shoulder joint. **a** Pig 10. The arrow points to the separate ossification centre or apophysis of the supraglenoid tubercle. There is a small osteochondrosis lesion labelled red cranially in the humeral head, near the bicipital groove. **b** Pig 45. There are apophyseal osteochondrosis lesions adjacent and deep to the ossification centre for the supraglenoid tubercle, labelled green. There is an uncommonly large lesion caudally in the humeral head, labelled red and possibly representing septic vascular failure and osteomyelitis, see Discussion. **c** Pig 6. There is a bilobed osteochondrosis lesion in the glenoid cavity, labelled green. **d** Pig 3. There is a cyst-like osteochondrosis lesion in the glenoid cavity, labelled green

All lesions observed in the shoulder joint were restricted to the two regions included in the labelling application. The supraglenoid tubercle ossifies from a separate centre or apophysis (Fig. 2a). Lesions were observed superficial and deep to this ossification centre (Fig. 2b) and were labelled with glenoid cavity lesions (Figs. 2c-d), which therefore comprised both articular and apophyseal osteochondrosis. Humeral lesions included cranial lesions near the bicipital groove predilection site (Fig. 2a) and lesions more caudally in the humeral head (Fig. 2b). The majority of humeral lesions were small, but five shoulder joints (1.2%) contained uncommonly large lesions (Fig. 2b) that spanned > 1000 labelled voxels. The possibility that the large lesions represented vascular failure due to sepsis and concomitant osteomyelitis is discussed below.

### Elbow joint

**Fig. 3:**
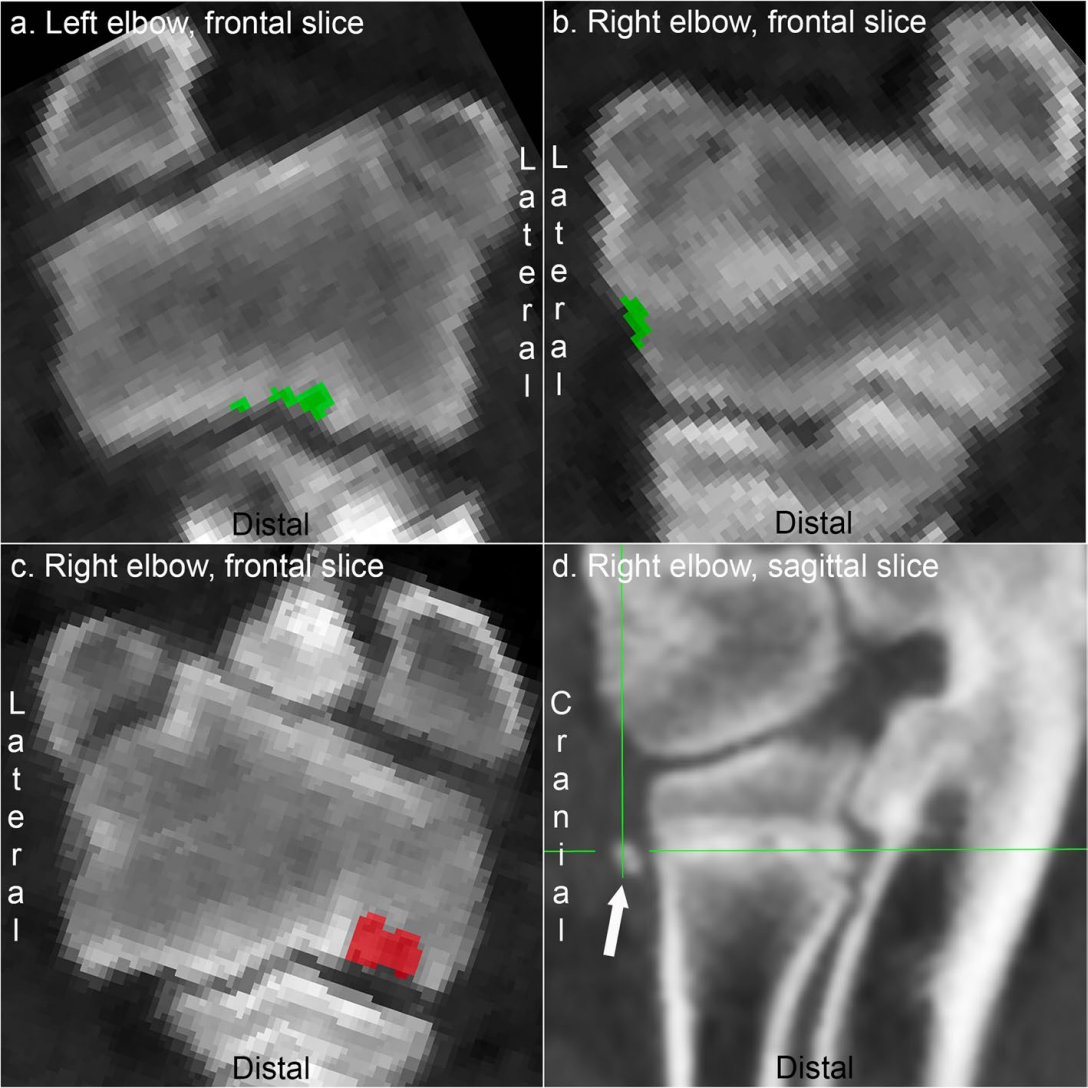
Labelling of lesions in the elbow joint. **a** Pig 2. There is a multi-lobulated osteochondrosis lesion at the sagittal ridge of the lateral condylar region, labelled green. **b** Pig 20. There is a small osteochondrosis lesion at the collateral ligament fossa, labelled green. **c** Pig 6. There is a cyst-like osteochondrosis lesion in the medial humeral condyle, labelled red. **d** Pig 31. The arrow points to a mineral hyperdense body adjacent to the proximal radial physis; a previously unreported location for such perichondrial new bone formation, but the lesion is not labelled because it represent physeal, rather than articular osteochondrosis. (Fig. 3d appears smoother because it was exported from the original CT scan via free software [https://horosproject.org/], rather than from the labelling application)

The majority of changes observed in the elbow joint were restricted to the four regions included in the labelling application. The lateral humeral condylar region included lesions at the sagittal ridge (Fig. 3a), capitulum and collateral ligament fossa (Fig. 3b) predilection sites, although lesions at the latter were relatively rare. Lesions in the medial humeral condyle were frequently cyst-like (Fig. 3c). Changes compatible with fragmented coronoid process were observed in a single elbow joint, but these were not labelled because the application did not include any ulnar regions. Mineral hyperdense bodies were observed adjacent to the proximal radial physis (Fig. 3d) in a low number of pigs, noted because the proximal radius represents a previously unreported location for such perichondrial new bone formation (see Discussion), but excluded from labelling because they represent physeal, rather than articular osteochondrosis.

### Hock joint

**Fig. 4:**
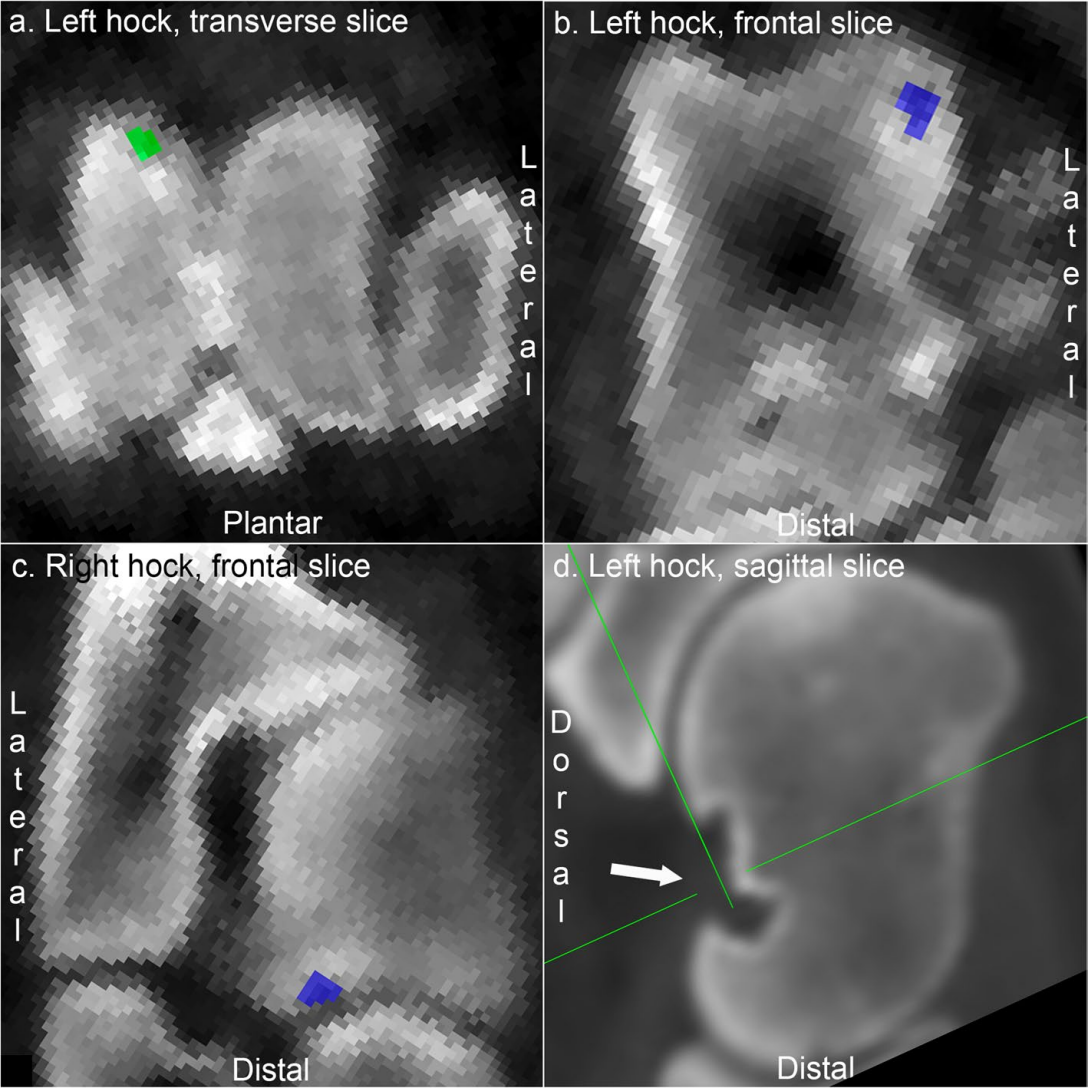
Labelling of lesions in the hock joint. **a** Pig 5. There is an osteochondrosis lesion proximo-dorsally in the medial trochlear ridge of the talus, labelled green. **b** Pig 10. There is a cyst-like osteochondrosis lesion proximally in the lateral trochlear ridge, labelled blue. **c** Pig 8. There is an osteochondrosis lesion disto-laterally in the caput of the talus, labelled blue. **d** Pig 31. The arrow points to a large, irregular and cyst-like defect at the collum of the talus. The defect has deliberately not been labelled as a lesion because there is currently no validated size threshold for distinguishing between normal nutrient foraminae and lesions in this location. (Fig. 4d appears smoother because it was exported from the original CT scan via free software [https://horosproject.org/], rather than from the labelling application)

All lesions in the hock joint were restricted to the three regions included in the labelling application. In both the lateral and medial halves of the talus, lesions were observed and labelled proximally in the trochlear ridges (Figs. 4a-b) and distally in the caput (Fig. 4c). Focal, hypodense areas were also observed in the dorsal midline at proximo-distal mid-height, i.e., at the collum of the talus (Fig. 4d). The majority of these areas were small, tubular and compatible with normal nutrient artery foraminae, but approximately 20–25% of the areas were large, irregular and cyst-like (Fig. 4d), potentially compatible with osteochondrosis lesions. The cyst-like defects were deliberately not labelled as lesions because there is currently no validated size threshold for distinguishing between normal nutrient foraminae and lesions in this location (see Discussion).

The labels for all joints in the first 10 pigs are summarised in Supplemental Table 1, to serve as an example of how the labelling process could apply to individual pigs.

### Quantitative results

The method resulted in labelling 6250 osteochondrosis lesions of 211,721.83 mm^3^ of total volume. The number of lesions ranged from 208 in the hock to 4306 in the stifle joint, as detailed in Table 1. Mean volume per lesion ranged from 26.21 mm^3^ in the shoulder to 42.06 mm^3^ in the elbow joint (Table 1). All pigs had at least one lesion in each femur, thus the per-pig prevalence of osteochondrosis was 100%. The per-joint prevalence of osteochondrosis, defined as one or both joints in a pair of joints being affected, ranged from 64.7% in the hock to 100% in the stifle joint (Table 1).

**Table 1:** Number of lesions and volume per lesion, per joint

Joint	Number of lesions	Number of affected joints of 402 total joints	Number of lesions per affected joint	Mean volume per lesion (mean of means for all regions in joint, see Table 2)	Number of affected joint pairs of 201 total pairs	Per-joint prevalence
Stifle	4306	402	10.71	26.55 mm^3^	201	100%
Shoulder	1184	371	3.19	26.21 mm^3^	198	98.5%
Elbow	552	258	2.14	42.06 mm^3^	165	82.1%
Hock	208	177	1.18	39.31 mm^3^	130	64.7%

The least-affected anatomical region was the medial half of the radial head which was the site of one lesion, whereas the most frequently-affected region was the lateral femoral condyle which was the site of 1797 lesions (Table 2). Mean volume per lesion was smallest at the distal intermediate ridge of the tibia and largest at the medial femoral condyle (Table 2).

**Table 2:** Number of lesions and volume per lesion, per anatomical region

Anatomical region	Number of lesions	Number of affected regions	Mean volume per lesion
Lateral femoral condyle	1797	371	18.55 mm^3^ (SD 51.85)
Medial femoral condyle	1576	400	63.64 mm^3^ (SD 133.85)
Glenoid cavity	976	358	29.24 mm^3^ (SD 64.54)
Medial femoral trochlear ridge	532	276	12.39 mm^3^ (SD 31.19)
Lateral femoral trochlear ridge	401	225	11.61 mm^3^ (SD 49.26)
Lateral humeral condyle	298	184	37.35 mm^3^ (SD 90.96)
Medial humeral condyle	245	154	45.17 mm^3^ (SD 122.79)
Humeral head	208	135	23.18 mm^3^ (SD 101.71)
Medial half of talus	149	136	50.24 mm^3^ (SD 55.50)
Lateral half of talus	57	53	59.72 mm^3^ (SD 84.83)
Lateral radial head	8	7	42.88 mm^3^ (SD 69.58)
Distal intermediate ridge of tibia	2	2	7.97 mm^3^ (SD 5.35)
Medial radial head	1	1	42.84 mm^3^ (SD not applicable)

### Clinical significance

As described in Methods, reference data are only available for the medial femoral condyle, where 311/1576 lesions (11.74%) were above the predicted critical size of 204.32 mm^3^, indicating a potential clinical significance.

### Correlation of lesion number and volume

**Fig. 5:**
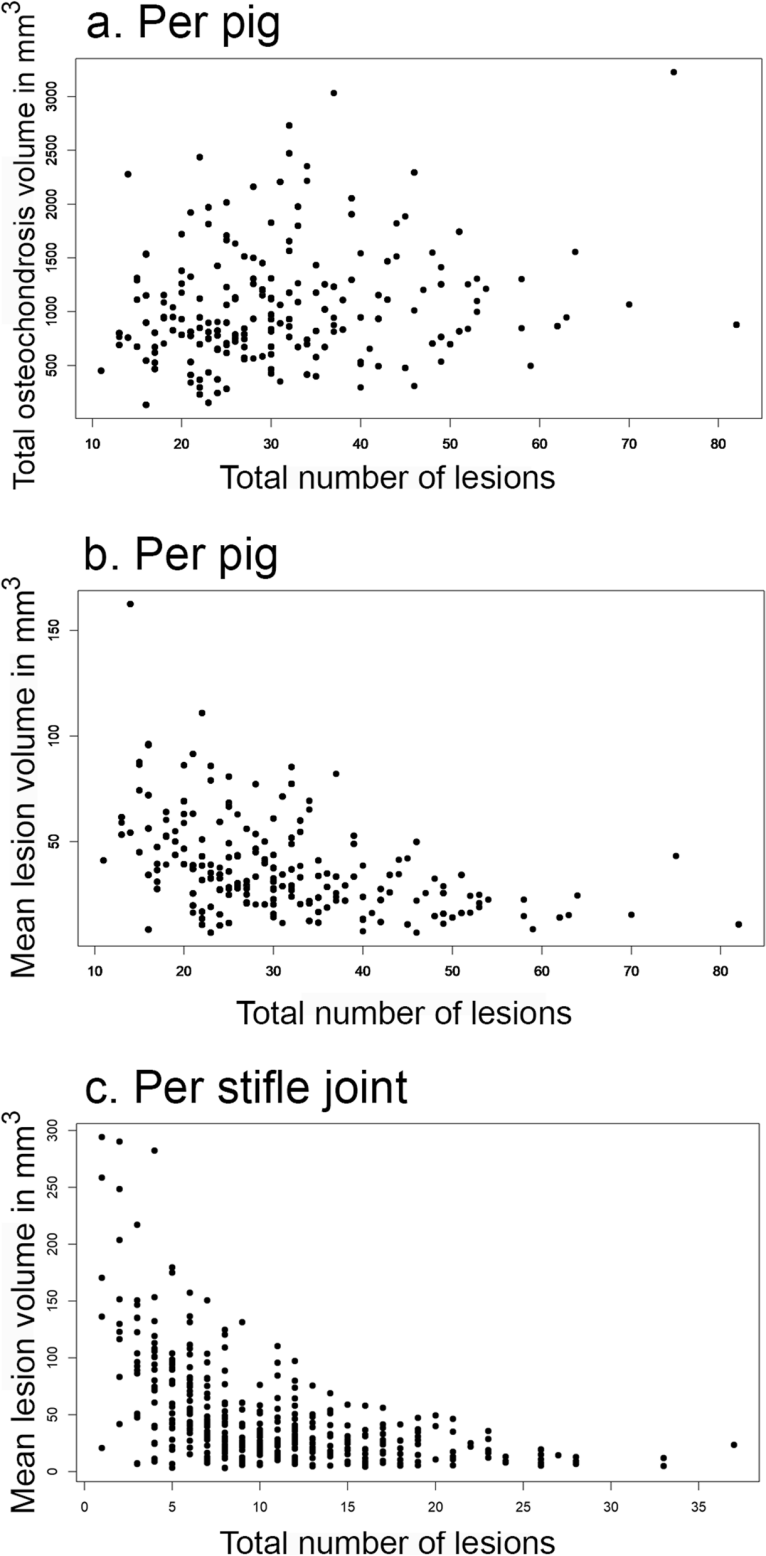
Volume of osteochondrosis versus total number of lesions in the 201 pigs. **a** Total volume of osteochondrosis per pig and **b** Mean lesion volume per pig; each black dot in **a.** and **b.** represents an individual pig. **c.** Mean lesion volume per stifle joint; each black dot represents an individual stifle joint

Individual pigs had a mean of 31.09 lesions (SD 12.51) and a mean volume of 1053.34 mm^3^ (SD 540.00) of osteochondrosis. Total volume of osteochondrosis versus total lesion number is shown in Fig. 5a. As the number of lesions increased, mean volume per lesion (Fig. 5b) tended to decrease. The pigs with the highest number of lesions had small lesions, whereas pigs with few lesions frequently had large lesions. It was not considered appropriate to group joints together because they had different mean lesion volumes, and there was minimal variance in lesion number in the shoulder, elbow and hock joints (Table 2). Correlation was therefore only tested for the stifle joint (Fig. 5c), where lesion number was moderately negatively correlated with mean volume per lesion at r (199df) = − 0.54, *p* < 0.001 (t = − 9.12).

## Discussion

The main outcome of this study was description of a method for labelling lesions in CT scans that also generated some new observations on osteochondrosis.

### Qualitative observations

In the current study, it was not possible to histologically validate the labelled lesions, but the authors previously validated identical CT lesions in the stifle [7] and hock joints [17]. Lesions detected by CT have not been validated in the shoulder or elbow joints previously, but lesions identified in this study appear to correspond to histologically validated shoulder and elbow lesions detected by macroscopic or radiographic evaluation of intact bones and slabs [1, 9, 18]. The positive predictive value of CT for the diagnosis of stifle osteochondrosis was reported to be 100% (95% confidence interval: 90–100) [7], but diagnosis is still subject to error. The majority of false-negative errors are due to failure to detect osteochondrosis latens lesions [7] and lesions that resolved before, or arose after the CT scan [8]. False-positive diagnosis can occur if normal features are interpreted as lesions. Most irregularities associated with normal growth are diffuse, gradual and peripheral, but some anatomical features are focal and sharply demarcated [17], similar to osteochondrosis lesions [7]. To date, one strategy to limit false-positive diagnosis has been to avoid evaluating central, midline or mid-height defects because this is where normal nutrient foraminae, synovial fossae and intertrochlear indentations tend to be located [17]. However, cyst-like defects were currently observed at the collum of the talus (Fig. 4d), and chondrocyte necrosis was previously detected at the margins of intertrochlear indentations [17]. Both observations indicate that lesions sometimes occur in the same sites as anatomical features. Thus, the strategy of avoiding evaluation of central, midline and mid-height defects to prevent false positive findings is becoming increasingly tenuous. Ideally, size limits for anatomical features should be established, allowing labelling of central, midline and mid-height defects, like the current talar cysts, as lesions if they are above the size thresholds for normal features.

A second form of false-positive error can occur if lesions are interpreted as heritably predisposed vascular failure and osteochondrosis, when they actually represent septic vascular failure. Bacterial thrombosis [19, 20] of cartilage canal vessels can trigger the same sequence of events as aseptic vascular failure in heritably predisposed osteochondrosis [21, 22]. Five of the evaluated shoulder joints had uncommonly large lesions, including pig 45 in Fig. 2b. Some of these pigs, including pig 45 could have two separate diseases: heritably predisposed osteochondrosis in the glenoid cavity, and acquired osteomyelitis in the humeral head. Alternatively, in pig 45, bacteria in the circulation may have caused septic, osteochondrosis-like lesions in the glenoid cavity, and osteomyelitis in the humeral head [22]. Heritably predisposed and septic vascular failure can affect the same anatomical structures; the cartilage canals [3, 4, 19, 20], causing lesions of identical appearance [23] that cannot be distinguished using CT scans. In horses, there is some indication that individuals with septic osteochondrosis-like lesions have more affected joints, and more lesions per affected joint than individuals with heritably predisposed osteochondrosis [24]. In the current study, there were 1184 shoulder lesions and only five of them were > 1000 voxels, suggesting that heritably predisposed vascular failure leads to lesions well below 1000 voxels, whereas septic vascular failure and osteomyelitis is likely associated with lesions > 1000 voxels, but this is merely a hypothesis at present. Work is underway trying to establish criteria to distinguish between septic and heritably predisposed vascular failure in live animals, to ensure that individuals with septic osteochondrosis-like lesions are not eliminated from the breeding stock [22].

The observation of cyst-like lesions in the proximal tibia (Fig. 1d) was new to us in pigs, but familiar in the sense that they appear similar to cysts previously described by Jeffcott & Kold [25] in the proximal tibia of young horses. Heritably predisposed vascular failure has been associated with vessels traversing tissue junctions like the ossification front [26, 27] and the perichondrial-growth cartilage junction where ligaments also attach; the latter was first documented at the caudal cruciate ligament in pig femurs [28]. The proximal tibial lesions observed here near the attachments of the cruciate and meniscal ligaments can probably be grouped together with lesions described at the origin of the long digital extensor tendon [7] as representations of vascular failure at ligament or tendon attachments [28]. Similarly, the proximal radial physis represents a new location for perichondrial new bone formation (Fig. 3d), previously described at the supraglenoid tubercle [29], and the distal fibular [29] and distal ulnar physes [30].

### Quantitative results

Although the volume of physeal osteochondrosis lesions in CT scans has been reported before [31], this is the first time that the CT volume of a large number of articular osteochondrosis lesions is reported. Our findings lay the foundation for future projects aiming to analyse the relationship between lesion volume and heritable predisposition, growth rate, conformation and other factors.

The existing CT screening for osteochondrosis can be described as evaluating eight anatomical regions as positive or negative for osteochondrosis [12]. With automated diagnosis, it will be possible to both count lesion number and quantify lesion volume. In the current study, pigs with many lesions tended to have smaller lesions than pigs with fewer lesions (Fig. 5). Indeed, our results obtained from the stifle joint suggest that a negative correlation may exist between lesion volume and number. This is important because large lesions are more likely to become clinically significant [32–34], indicating a need for evaluation of both lesion number and volume during breeding selection.

The observation that mean lesion volume tended to be smaller in proximal than distal joints (Table 1) was unexpected. Proximal joints have thicker growth cartilage than distal joints at the same age [35], therefore proximal joints were expected to have larger lesions, following vascular failure. One possible explanation for the larger size of lesions found in distal joints is that osteochondrosis develops earlier in distal than in proximal joints [8]. Lesions may therefore have been of similar size, but appeared larger in distal joints because they were more completely surrounded and outlined by bone at the age of the CT examination [6]. An alternative explanation is that growth cartilage also has more vessels in proximal than distal joints at a given age [27, 35, 36], meaning diffusion distance to intact, adjacent vessels may actually be shorter in proximal, compared to distal joints. This assumption can be tentatively refuted by comparing current observations with previous perfusion studies [3, 4]. In the current study, mean lesion volume was 3.4 times greater in the medial than the lateral femoral condyle (Table 2), despite findings from previous perfusion studies demonstrating a greater number of vessels present in the medial, than the lateral condyle at the same age [3, 4]. Chondrocytes in the lateral condyle therefore do not have better access to collateral supply, and this does not explain the observed differences in lesion volume. A third possible explanation is that at the ages of 13 and 15 weeks, many of the vessels in the lateral femoral condyle are short stumps with few side branches, meaning they each supply a smaller volume of growth cartilage compared to the long vessels with many side branches observed in the medial femoral condyle at the same ages [4]. Thus, the observed differences between small and large lesions may be explained by the length and branching of the vessels that failed being different to begin with. However, this may not be the correct explanation for the observed differences between the femoral condyles, because the chondrocytes around the vessel stumps in the lateral condyle are so close to the ossification front [4] that they are likely to survive by diffusion [3], even if the vessel stump should fail.

In addition to anatomical features, lesion volume may also be influenced by physiological differences that are difficult to study. For example, peri-canalicular chondrocytes undergo coagulative necrosis in 3–14 days, whereas inter-canalicular chondrocytes undergo necrosis in 7–21 days following vascular failure [3, 5, 6] and this has been attributed to peri-canalicular chondrocytes having higher oxidative metabolism [37]. Portions of lesions that become surrounded by the ossification front in < 14 days are therefore smaller than portions that become surrounded in > 21 days [6]. We believe that the observed differences in lesion volume will ultimately be explained by the configuration of the vessels that fail, cartilage thickness, the metabolic activity of chondrocytes and rate of advancement of the ossification front. We intend to pursue this by analysing the shape and dimensions of the current CT lesion masks further. The metabolism of medial versus lateral femoral chondrocytes could also be studied [37], or lesions could be compared using three-dimensional modalities that enable simultaneous visualisation of vessel configuration, osteochondrosis latens and manifesta lesions, such as second harmonics generation microscopy [28], or various magnetic resonance imaging sequences [38, 39].

### Clinical significance

None of the examined pigs had OCD lesions, or clinical signs of osteochondrosis. Pigs with disease that do not respond to medical treatment are removed, and therefore do not make it to the CT scan at the end of the boar test. It is also possible that progression of osteochondrosis manifesta to OCD requires higher levels of physical activity than the relative confinement of conventional housing units [40]. When as many as 6250 lesions were identified, it felt important to estimate what portion was likely to persist and progress to clinically significant disease. Critical defect size [33, 34] is a highly unsatisfactory measure because it is reported as the absolute diameter of a cylindrical defect, when it really needs to be quantified relative to the size of the femur it is located in [41], but it is the only evidence-based measure available to us. Comparison with critical defect size indicated that in the region of ~ 12% of the current medial femoral condylar lesions could have become clinically significant [33, 34]. In future studies, critical defect size can be determined for commercial pigs, by quantifying the volume of lesions in CT scans then monitoring which lesions progressed to OCD after the physical activities associated with transport to slaughter in boars that were not selected for breeding [42].

## Conclusions

The described labelling method may serve as a foundation for the development of automated diagnosis of osteochondrosis manifesta and cyst-like lesions by machine learning in future. Both lesion number and volume should be considered during breeding selection, if feasible. The apparent inverse relationship between lesion number and volume warrants further investigation.

## Methods

### Pigs

CT scans, age and weight of boars were retrospectively extracted from boar test archives. The study was approved by the Ethical Committee of the Norwegian University of Life Sciences Faculty of Veterinary Medicine, reference number 14/04723–68.

Data analysis was completed for 201 boars. The pigs were balanced for four breeds: Landrace, Duroc, Synthetic A and B breeds. Included Landrace and Duroc boars were scanned between 2016 and 2020, whereas Synthetic A and B boars were scanned between 2018 and 2020. The four breed groups were treated as a single study population.

### CT-scanning

Landrace and Duroc boars were scanned in a 32-slice CT-scanner (LightSpeed Pro32, GE Healthcare, Chicago, Illinois, USA) and Synthetic A and B boars were scanned in a 64-slice CT-scanner (Revolution Evo, GE Healthcare, Chicago, Illinois, USA). The scanning procedure has been described before [13], but briefly: boars were sedated and placed in sternal recumbency with free limb position. The field of view was collimated from the snout to whichever was most caudal of the tail or hocks of the pig, and scan parameters were 120 kV, dynamic mA up to 400, slice thickness of 1.25 mm and pitch 1.

### Preparation of scans for labelling

**Fig. 6:**
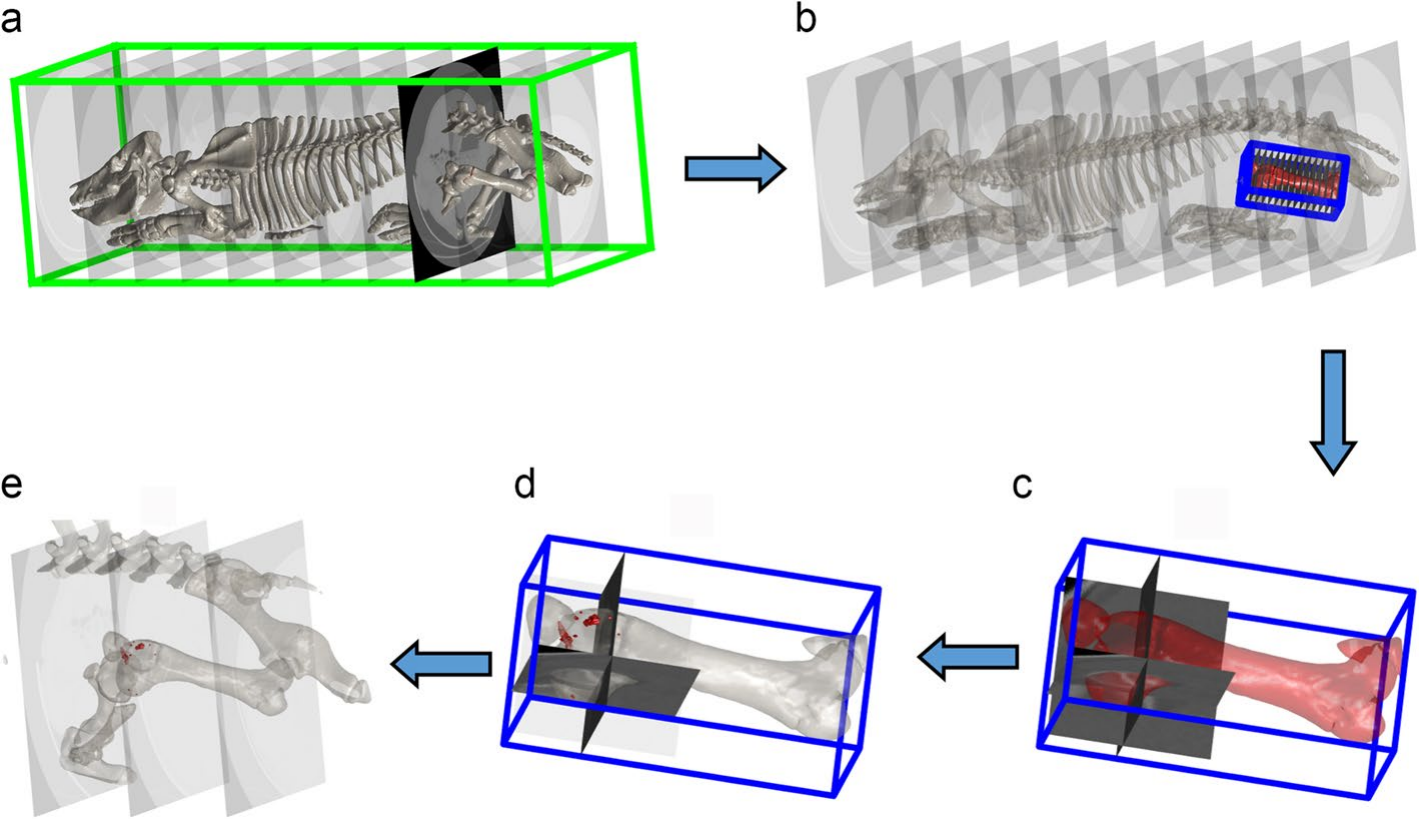
Preparation of CT scans for labelling. **a** The original scan (green box) was treated as an array of ~ 1000 slices with 512 × 512 pixels. **b-c** A new scan volume (blue box) was constructed around the femur based on image moment invariants. The grid size for the new femur scan volume was 300 × 150 × 150 voxels. **c-d** The distal end of the new volume was used to extract a volume containing the stifle joint, where transverse plane images were perpendicular to the long axis of the femur, irrespective of limb position in the original scan (**a**). **d-e** The x, y and z coordinates from the original CT scan remained associated with the new voxels, meaning applied labels (red areas) could be translated back to their location within the original scan

Scans were prepared for labelling using MatLab (v. 9.9.0 [R2020b], The MathWorks Inc., Natick, Massachusetts, USA). The CT scan was treated as an array of ~ 1000 slices with 512 × 512 pixels; the total slice number varied somewhat with the length of the individual pig (Fig. 6a). An artificial intelligence method previously described by Kvam et al. [43] was used to segment the major limb bones. Segmentations were visually inspected and improved by manual adjustment of the two-dimensional masks where necessary.

New scan volumes were constructed around the humerus, femur (Fig. 6b) and tibia based on their image moment invariants [44], see Gangsei et al. [45] for details. The grid sizes for the new volumes were 256 × 128 × 128 voxels for the humerus, 300 × 150 × 150 voxels for the femur (Fig. 6c) and 300 × 128 × 128 voxels for the tibia. Because grid sizes were fixed, voxel resolution varied slightly with the scaling of individual bones, but resolution from the original scan remained associated with each new volume. The proximal and distal ends of the new volumes were used to extract volumes containing the shoulder, elbow, stifle (Fig. 6d) and hock joints for labelling. The new volumes contained transverse images that were perpendicular to the long axis of the humerus, femur (Fig. 6d) or tibia, irrespective of limb position in the original scan (Fig. 6a). The re-stacking of voxels results in some regular irregularities, apparent as stripes in the images, but these were readily distinguishable from focal lesions. The new volumes also contained two planes that were variably aligned with the frontal and sagittal planes, but always orthogonal to the transverse plane and each other. The x, y and z coordinates from the original CT scan remained associated with the new voxels, meaning applied labels could be transferred back to their location within the original CT scan (Fig. 6e).

### Labelling application

An application for manual image labelling was developed using MatLab. The application showed all three image planes and had functions for adjusting window width/level, smoothing, scrolling, zooming and correlating image planes using crosshairs. The application also had tools for labelling lesions by clicking on individual pixels or drawing around larger areas to create so-called “masks”. Different colours were used to represent different categories of observations, described below. Once a pixel was labelled with a particular colour in one image plane, it immediately showed up as the same colour in the two orthogonal planes. Each labelled pixel within a mask could then be exported for further analyses as a unit containing information about its colour and x, y and z coordinates, i.e., a classified voxel, from which information about its relationship to neighbouring voxels could also be extracted.

### Labelling strategy

Scans were labelled by a veterinary radiologist with 14 years of experience who was blinded to the identity of the pig. A strategy was pilot-tested where different colours represented categories of qualitative change, e.g., primary osteochondrosis defect, secondary marginal sclerosis etc., but it was difficult to label consistently when individual lesions required multiple colours, and the strategy generated few observations spread on many categories unsuitable for machine learning and was therefore abandoned.

Instead, a definitive strategy was adopted where only primary osteochondrosis defects were labelled as lesions (Figs. 1, 2, 3 and 4) [7], and where different colours represented different anatomical regions. Thirteen anatomical regions were used, comprising the glenoid cavity, humeral head, medial and lateral humeral condyles, medial and lateral halves of the proximal radius, medial and lateral trochlear ridges of the femur, medial and lateral condyles of the femur, distal intermediate ridge of the tibia and medial and lateral halves of the talus. Stifle joints were labelled first, followed by the shoulder, elbow and hock joints.

### Parameters observed

Voxels were labelled as osteochondrosis lesions if they were located within focal, sharply demarcated and uniformly hypodense, single or multi-lobulated (“stair-step”) defects in or near the ossification front (Fig. 2c). Defects in the ossification front have previously been histologically validated to represent the osteochondrosis manifesta stage of the disease [7, 17], whereas spherical defects that are deeper than manifesta lesions have been validated to represent subchondral pseudo- or true bone cysts [7, 46]. It is not possible to distinguish between true and pseudocysts in CT scans, so such lesions will be referred to as “cyst-like”. Osteochondrosis latens and dissecans stages were not labelled, thus there was no risk of confusion and the labelled osteochondrosis manifesta lesions will be referred to simply as osteochondrosis lesions.

The following changes were not labelled: secondary responses, physeal osteochondrosis lesions, hypodense channels or foci presumed to represent nutrient artery foraminae, synovial fossae or intertrochlear indentations based on their typical location in the centre, midline and at mid-height of epiphyses or the talus [17].

### Lesion number

Lesion masks that were not connected at their faces, edges or corners were counted as separate lesions, as determined using the 26-connected version of a MatLab tool for analysing three-dimensional voxel connectivity (https://se.mathworks.com/help/images/pixel-connectivity.html). Only masks that contained four or more connected voxels were counted as lesions, to ensure that lesions spanned at least two voxels in one of the three image planes, thus reducing the risk of labelling normal contour irregularities as lesions.

Both single-lobe and multi-lobulated lesions were counted as one lesion.

### Lesion size

Lesion size was defined as the volume of an osteochondrosis or cyst-like lesion that was outlined by bone. The superficial margin of osteochondrosis lesions could not be visualised because it bordered on iso-dense cartilage. By default, the labelled area was therefore extended until it was level with the adjacent, normal ossification front.

### Clinical significance

Pigs that develop disease are removed from the boar test, so included pigs did not have clinical signs of osteochondrosis. An attempt was nevertheless made at suggesting the portion of lesions that might progress to clinical disease through comparison with critical defect size, defined as the size above which defects will not heal without intervention [33]. Studies to determine critical defect size are only available for miniature pigs [33]. Critical defect size was translated from miniature to the more regularly-sized, commercially reared pigs that enter the boar test as follows: Chang et al. [34] indicated that the diameter of critical-sized defects was 38.1% of the width of the medial femoral condyle. This width was measured to approximately 22,5 mm for the current included pigs, and 38.1% of that or ≈8.6 mm was used as the diameter for the translated cylindrical defect. Based on Ahern et al. [33], the depth of the defect was set to 6 mm, resulting in a total cylinder volume of 346.30 mm^3^. According to Ahern et al. [33], the portion of critical-sized defects that is located within bone and therefore detectable by CT is 59%, resulting in a final translated critical-sized bone defect of 204.32 mm^3^ for the medial femoral condyle of commercial pigs.

### Agreement

Intra-observer agreement was informally tested by labelling all regions in 10 pigs two times up to 7 months apart (Supplemental Table 2). This generated 260 paired observations, with agreement on whether a region was positive or negative on 213 occasions (81.9%). Disagreement was handled by keeping the first labelling.

### Correlations

Correlations were tested using Pearson’s correlation coefficient with the level of significance set at < 0.05.

## Supplementary Information

**Additional file 1: Supplemental Movie 1.**

**Additional file 2: Supplemental Movie 2. Supplemental Movies 1 and 2** show labelling of osteochondrosis lesions in the right distal femur of pig 93. There is a large lesion in the medial femoral condyle (large group of connected green dots), and smaller lesions (smaller groups of green dots) in the medial condyle and both trochlear ridges. **Movie 1** shows two-dimensional images representing transverse, frontal and sagittal planes through the medial femoral condylar lesion. The smaller lesions are out of the shown planes of section, but correctly positioned in space. **Movie 2** includes a three-dimensional surface rendering of the mineral density of the femur where the location of the smaller lesions can be seen.

**Additional file 3: Supplemental Table 1.** Annotations in the first 10 pigs.

**Additional file 4: Supplemental Table 2.** Intra-observer agreement on whether an anatomical region was positive or negative for osteochondrosis.

## Data Availability

The datasets analysed in the current study are not publicly available because they belong to a commercial pig breeding company and making them publicly available conflicts with company policy, but data are available from the corresponding author on reasonable request.

## References

[1] Reiland S (1978). Morphology of osteochondrosis and sequelae in pigs. Acta Radiol Suppl.

[2] Ytrehus B, Carlson CS, Ekman S (2007). Etiology and pathogenesis of osteochondrosis. Vet Pathol.

[3] Carlson CS, Meuten DJ, Richardson DC (1991). Ischemic necrosis of cartilage in spontaneous and experimental lesions of osteochondrosis. J Orthop Res.

[4] Ytrehus B, Carlson CS, Lundeheim N, Mathisen L, Reinholt FP, Teige J, Ekman S (2004). Vascularisation and osteochondrosis of the epiphyseal growth cartilage of the distal femur in pigs--development with age, growth rate, weight and joint shape. Bone.

[5] Ytrehus B, Andreas Haga H, Mellum CN, Mathisen L, Carlson CS, Ekman S, Teige J, Reinholt FP (2004). Experimental ischemia of porcine growth cartilage produces lesions of osteochondrosis. J Orthop Res.

[6] Olstad K, Hendrickson EHS, Carlson CS, Ekman S, Dolvik NI (2013). Transection of vessels in epiphyseal cartilage canals leads to osteochondrosis and osteochondrosis dissecans in the femoro-patellar joint of foals; a potential model of juvenile osteochondritis dissecans. Osteoarthr Cartil.

[7] Olstad K, Kongsro J, Grindflek E, Dolvik NI (2014). Ossification defects detected in CT scans represent early osteochondrosis in the distal femur of piglets. J Orthop Res.

[8] Olstad K, Kongsro J, Grindflek E, Dolvik NI (2014). Consequences of the natural course of articular osteochondrosis in pigs for the suitability of computed tomography as a screening tool. BMC Vet Res.

[9] Grøndalen T (1974). Osteochondrosis and arthrosis in pigs. I. Incidence in animals up to 120 kg live weight. Acta Vet Scand.

[10] Reiland S (1978). Pathology of so-called leg weakness in the pig. Acta Radiol Suppl.

[11] Heinonen M, Oravainen J, Orro T, Seppa-Lassila L, Ala-Kurikka E, Virolainen J, Tast A, Peltoniemi OA (2006). Lameness and fertility of sows and gilts in randomly selected loose-housed herds in Finland. Vet Rec.

[12] Aasmundstad T, Kongsro J, Wetten M, Dolvik NI, Vangen O (2013). Osteochondrosis in pigs diagnosed with computed tomography: heritabilities and genetic correlations to weight gain in specific age intervals. Animal.

[13] Gjerlaug-Enger E, Kongsro J, Odegard J, Aass L, Vangen O (2012). Genetic parameters between slaughter pig efficiency and growth rate of different body tissues estimated by computed tomography in live boars of landrace and duroc. Animal.

[14] Mukherjee S, Nazemi M, Jonkers I, Geris L (2020). Use of computational modeling to study joint degeneration: a review. Front Bioeng Biotechnol.

[15] de Koning DB, van Grevenhof EM, Laurenssen BF, van Weeren PR, Hazeleger W, Kemp B (2014). The influence of floor type before and after 10 weeks of age on osteochondrosis in growing gilts. J Anim Sci.

[16] Zeder MA, Lemoine X, Payne S (2015). A new system for computing long-bone fusion age profiles in *Sus scrofa*. J Archaeol Sci.

[17] Etterlin PE, Ekman S, Strand R, Olstad K, Ley CJ (2017). Osteochondrosis, synovial fossae, and articular indentations in the talus and distal tibia of growing domestic pigs and wild boars. Vet Pathol.

[18] Woodard JC, Becker HN, Poulos PW (1987). Effect of diet on longitudinal bone growth and osteochondrosis in swine. Vet Pathol.

[19] Denecke R, Trautwein G, Kaup FJ (1986). The role of cartilage canals in the pathogenesis of experimentally induced polyarthritis. Rheumatol Int.

[20] Speers DJ, Nade SM (1985). Ultrastructural studies of adherence of Staphylococcus aureus in experimental acute hematogenous osteomyelitis. Infect Immun.

[21] Olstad K, Wormstrand B, Kongsro J, Grindflek E (2019). Osteochondrosis in the distal femoral Physis of pigs starts with vascular failure. Vet Pathol.

[22] Wormstrand B, Østevik L, Ekman S, Olstad K (2018). Septic arthritis/osteomyelitis may Lead to Osteochondrosis-like lesions in foals. Vet Pathol.

[23] Olstad K, Ekman S, Carlson CS (2015). An update on the pathogenesis of Osteochondrosis. Vet Pathol.

[24] Hendrickson EHS, Lykkjen S, Dolvik NI, Olstad K (2018). Prevalence of osteochondral lesions in the fetlock and hock joints of Standardbred horses that survived bacterial infection before 6 months of age. BMC Vet Res.

[25] Jeffcott LB, Kold SE (1982). Clinical and radiological aspects of stifle bone cysts in the horse. Equine Vet J.

[26] Ytrehus B, Ekman S, Carlson CS, Teige J, Reinholt FP (2004). Focal changes in blood supply during normal epiphyseal growth are central in the pathogenesis of osteochondrosis in pigs. Bone.

[27] Olstad K, Ytrehus B, Ekman S, Carlson CS, Dolvik NI (2008). Epiphyseal cartilage canal blood supply to the tarsus of foals and relationship to osteochondrosis. Equine Vet J.

[28] Finnøy A, Olstad K, Lilledahl MB (2017). Non-linear optical microscopy of cartilage canals in the distal femur of young pigs may reveal the cause of articular osteochondrosis. BMC Vet Res.

[29] Olstad K, Wormstrand B, Kongsro J, Grindflek E (2019). Computed tomographic development of physeal osteochondrosis in pigs. BMC Vet Res.

[30] Bittegeko SB, Arnbjerg J (1994). The sequelae of distal ulna physeal dyschondroplasia (osteochondrosis) lesions in breeding swine--a radiological investigation in Danish Landrace pigs. Zentralbl Veterinarmed A.

[31] Grez-Capdeville M, Gross N, Baker JC, Shutter JA, Haas AR, Wilson ME (2020). Alleged predisposing dietary factors fail to increase the incidence of osteochondrosis-like lesions in growing pigs at 14 and 24 wk of age. J Anim Sci..

[32] Bravo C, Kawamura H, Yamaguchi T, Hotokebuchi T, Sugioka Y (1996). Experimental osteochondritis dissecans--the role of cartilage canals in chondral fractures of young rabbits. Fukuoka Igaku Zasshi.

[33] Ahern BJ, Parvizi J, Boston R, Schaer TP (2009). Preclinical animal models in single site cartilage defect testing: a systematic review. Osteoarthr Cartil.

[34] Chang C-H, Hsu Y-M, Hsiao C-N, Kuo T-F, Chang M-H. Critical-sized osteochondral defects of young miniature pigs as a preclinical model for articular cartilage repair. Biomed Eng. 2014;26(01):1450003 1–8.

[35] Hendrickson EHS, Olstad K, Nødtvedt A, Pauwels E, van Hoorebeke L, Dolvik NI (2015). Comparison of the blood supply to the articular-epiphyseal growth complex in horse vs. pony foals. Equine Vet J.

[36] Olstad K, Ytrehus B, Ekman S, Carlson CS, Dolvik NI (2008). Epiphyseal cartilage canal blood supply to the distal femur of foals. Equine Vet J.

[37] Shapiro IM, Golub EE, Chance B, Piddington C, Oshima O, Tuncay OC, Frasca P, Haselgrove JC (1988). Linkage between energy status of perivascular cells and mineralization of the chick growth cartilage. Dev Biol.

[38] Toth F, Nissi MJ, Zhang J, Benson M, Schmitter S, Ellermann JM, Carlson CS (2013). Histological confirmation and biological significance of cartilage canals demonstrated using high field MRI in swine at predilection sites of osteochondrosis. J Orthop Res.

[39] Ludwig KD, Johnson CP, Zbyn S, Nowacki A, Marette S, Takahashi T, Macalena JA, Nelson BJ, Tompkins MA, Carlson CS (2020). MRI evaluation of articular cartilage in patients with juvenile osteochondritis dissecans (JOCD) using T2 * mapping at 3T. Osteoarthr Cartil.

[40] Etterlin P, Ytrehus B, Lundeheim N, Heldmer E, Osterberg J, Ekman S (2014). Effects of free-range and confined housing on joint health in a herd of fattening pigs. BMC Vet Res.

[41] Lee S, Frank RM, Christian DR, Cole BJ (2019). Analysis of defect size and ratio to condylar size with respect to outcomes after isolated osteochondral allograft transplantation. Am J Sports Med.

[42] Nakano T, Aherne FX (1988). Involvement of trauma in the pathogenesis of osteochondritis Dissecans in swine. Can J Vet Res.

[43] Kvam J, Gangsei LE, Kongsro J, Schistad Solberg AH (2018). The use of deep learning to automate the segmentation of the skeleton from CT volumes of pigs. Transl Anim Sci.

[44] Hu M-K (1962). Visual pattern recognition by moment invariants. IRE Trans Inf Theory.

[45] Gangsei LE, Kongsro J, Olstad K, Grindflek E, Sæbø S (2016). Building an *in vivo* anatomical atlas to close the phenomic gap in animal breeding. Comput Electron Agric.

[46] Olstad K, Østevik L, Carlson CS, Ekman S (2015). Osteochondrosis can lead to formation of pseudocysts and true cysts in the subchondral bone of horses. Vet Pathol..

