# Supplementary material for: A method for labelling lesions for machine learning and some new observations on osteochondrosis in computed tomographic scans of four pig joints
Source: BMC Vet Res. 2022 Aug 31;18:328. doi: 10.1186/s12917-022-03426-x (PMC9429582; doi:10.1186/s12917-022-03426-x)
Supplement: Supplementary file 3 — Additional file 3: Supplemental Table 1. Annotations in the first 10 pigs. [file 12917_2022_3426_MOESM3_ESM.docx]

**Supplemental Table 1.** Annotations in the first 10 pigs

|  |  | **Stifle joint** | | | | **Shoulder joint** | | **Elbow joint** | | | | **Hock joint** | | |
| --- | --- | --- | --- | --- | --- | --- | --- | --- | --- | --- | --- | --- | --- | --- |
| **Pig number** | **Side** | **Medial trochlear ridge** | **Medial femoral condyle** | **Lateral trochlear ridge** | **Lateral femoral condyle** | **Glenoid cavity** | **Humeral head** | **Medial humeral condyle** | **Medial radial head** | **Lateral humeral condyle** | **Lateral radial head** | **Distal intermediate ridge of tibia** | **Medial half of talus** | **Lateral half of talus** |
| 1 | Left | - | + | - | + | +^1^ | - | - | - | - | - | - | - | - |
|  | Right | + | + | + LDE^2^ | + | + | - | - | - | - | - | - | + pr^3^ | - |
| 2 | Left | - | + | - | - | + | - | - | - | + sr^4^ | - | - | + di^5^ | - |
|  | Right | - | + | - | + | + | - | + | - | + | - | - | - | - |
| 3 | Left | - | + | + | + | + cyst | - | - | - | + | - | - | - | - |
|  | Right | + | + cyst | + | + | - | - | - | - | + sr | - | - | + di | - |
| 4 | Left | + cyst | + | - | + | + apo^6^ | - | - | - | - | + | - | - | - |
|  | Right | + cyst | + | + | + | + apo | - | - | - | - | - | - | - | - |
| 5 | Left | + | + cyst | + | + | + | - | - | - | + sr | - | - | + pr-do^7^ | - |
|  | Right | + | + | - | + | + | - | - | - | + sr | - | - | + pr-ab^8^ cyst | - |
| 6 | Left | - | + | - | - | + bi-lobe^9^ | - | + cyst | - | - | - | - | - | - |
|  | Right | + | + | - | + | + | - | + bi-lobe/cyst | - | - | - | - | - | - |
| 7 | Left | + | + | - | + | + | - | - | - | + | - | - | - | - |
|  | Right | + | + | - | + | - | - | - | - | + | - | - | + di | + di |
| 8 | Left | + cyst | + | + | + | + | - | - | - | + | - | - | - | - |
|  | Right | + | + | - | + | + | + cranial | - | - | - | - | - | - | + di |
| 9 | Left | + | + | + | + | + | - | + cyst | - | - | - | - | - | - |
|  | Right | + | + | - | + | - | - | - | - | - | - | - | - | - |
| 10 | Left | + | + | - | + | + | - | - | - | - | - | - | - | + pr cyst |
|  | Right | + | + | - | + | + | + cranial | - | - | - | - | - | - | - |
| Sum |  | 15 | 20 | 7 | 18 | 17 | 2 | 4 | 0 | 9 | 1 | 0 | 6 | 3 |

^1^+: A plus sign denotes presence of a lesion in the given region but does not represent the number of lesions counted in that region. ^2^LDE: Lesion at the origin of the long digital extensor tendon in the lateral trochlear ridge. ^3^pr: Proximal end. ^4^sr: Sagittal ridge. ^5^di: Distal end. ^6^apo: Lesion at the ossification centre (apophysis) for the supraglenoid tubercle. ^7^do: Dorsal. ^8^ab: Abaxial. ^9^bi-lobe: Multi-lobulated defect comprising two lobes. Grey box: an image of the lesion is included in Figs. 1-4.
