# Supplementary material for: A method for labelling lesions for machine learning and some new observations on osteochondrosis in computed tomographic scans of four pig joints
Source: BMC Vet Res. 2022 Aug 31;18:328. doi: 10.1186/s12917-022-03426-x (PMC9429582; doi:10.1186/s12917-022-03426-x)
Supplement: Supplementary file 4 — Additional file 4: Supplemental Table 2. Intra-observer agreement on whether an anatomical region was positive or negative for osteochondrosis. [file 12917_2022_3426_MOESM4_ESM.docx]

**Supplemental Table 2.** Intra-observer agreement on whether an anatomical region was positive or negative for osteochondrosis

| **Pig number**  **and breed^1^** | **Left stifle** | **Right stifle** | **Left shoulder** | **Right shoulder** | | **Left elbow** | **Right elbow** | **Left hock** | **Right hock** | | **Agreement per pig** |
| --- | --- | --- | --- | --- | --- | --- | --- | --- | --- | --- | --- |
| 1  Synthetic B | 2/4 regions | 3/4 regions | 1/2 regions | 1/2 regions | | 3/4 regions | 4/4 regions | 1/3 regions | 3/3 regions | | 18/26 regions (69.2%) |
| 2  Synthetic A | 2/4 regions | 2/4 regions | 1/2 regions | 1/2 regions | | 4/4 regions | 4/4 regions | 3/3 regions | 3/3 regions | | 20/26 regions (76.9%) |
| 3  Synthetic A | 2/4 regions | 4/4 regions | 2/2 regions | 1/2 regions | | 4/4 regions | 4/4 regions | 3/3 regions | 3/3 regions | | 23/26 regions (88.5%) |
| 4  Synthetic B | 2/4 regions | 2/4 regions | 1/2 regions | 1/2 regions | | 4/4 regions | 4/4 regions | 3/3 regions | 3/3 regions | | 21/26 regions (80.8%) |
| 5  Synthetic A | 4/4 regions | 4/4 regions | 2/2 regions | 1/2 regions | | 4/4 regions | 4/4 regions | 3/3 regions | 3/3 regions | | 25/26 regions (96.2%) |
| 6  Duroc | 4/4 regions | 3/4 regions | 2/2 regions | 1/2 regions | | 3/4 regions | 4/4 regions | 2/3 regions | 3/3 regions | | 22/26 regions  (84.6 %) |
| 7  Duroc | 4/4 regions | 3/4 regions | 2/2 regions | 2/2 regions | | 3/4 regions | 3/4 regions | 3/3 regions | 3/3 regions | | 23/26 regions (88.5%) |
| 8  Landrace | 3/4 regions | 3/4 regions | 1/2 regions | 1/2 regions | | 3/4 regions | 3/4 regions | 3/3 regions | 3/3 regions | | 20/26 regions (76.9%) |
| 9  Duroc | 3/4 regions | 3/4 regions | 1/2 regions | 1/2 regions | | 3/4 regions | 4/4 regions | 3/3 regions | 3/3 regions | | 21/26 regions (80.8%) |
| 10  Landrace | 3/4 regions | 1/4 regions | 1/2 regions | 2/2 regions | | 4/4 regions | 4/4 regions | 3/3 regions | 2/3 regions | | 20/26 regions (76.9%) |
| Sum | 29/40 regions (72.5%) | 28/40 regions (70%) | 14/20 regions (70%) | 12/20 regions (60%) | | 35/40 regions (87.5%) | 37/40 regions (92.5%) | 27/30 regions (90%) | 29/30 regions (96.7%) | | 213/260 regions (81.9%) |
| Agreement per joint | 57/80 regions (71.3%) | | 26/40 regions (65%) | | 72/80 regions (90%) | | | 56/60 regions (93.3%) | |  | |

^1^Ten pigs were selected at random, including two or three pigs from each breed. The pig numbering in this table does not correspond to the numbering in the manuscript. Please see additional comments, below.

*Comments:*

-All stifle joints were labelled first, followed by the shoulder, elbow and hock joints.

-Variable agreement between individual pigs may reflect whether the pigs were scored early or late during the study, combined with observer experience and agreement improving during the study.

-Although anatomical regions and other factors were not identical, agreement was lower than previously reported (71.5%, reported in Olstad K, Kongsro J, Grindflek E, Dolvik NI: Ossification defects detected in CT scans represent early osteochondrosis in the distal femur of piglets. *J Orthop Res* 2014, 32(8):1014-1023) for the same observer in the stifle and shoulder joints.

-Lower agreement in the shoulder joint is believed to reflect the fact that lesions at the ossification centre/apophysis for the supraglenoid tubercle may have been handled inconsistently at the start of the study.

-Lower agreement in the stifle joint probably reflects that the observer attempted to label smaller lesions than before, in the interest of subsequent machine learning performing as well as possible.

-High agreement in the elbow and hock joints may reflect that observer experience and agreement improved during the study.

-High agreement in the elbow and hock joints is also believed to reflect the fact that mean volume per lesion was larger in the elbow and hock, than the stifle and shoulder joints, and it was easier to agree on the presence of large than small lesions.
